# PSMD7 downregulation induces apoptosis and suppresses tumorigenesis of esophageal squamous cell carcinoma *via* the mTOR/p70S6K pathway

**DOI:** 10.1002/2211-5463.12394

**Published:** 2018-03-07

**Authors:** Ke Shi, Jin‐zhong Zhang, Rui‐li Zhao, Liang Yang, Dan Guo

**Affiliations:** ^1^ Department of Biochemistry and Molecular Biology Henan Medical College China; ^2^ Editorial Department of Journal of Henan University of Technology Henan University of Technology Zhengzhou China; ^3^ College of Biological Engineering Henan University of Technology Zhengzhou China; ^4^ Department of Microbiology and Immunology and Medicine Henan Medical College China

**Keywords:** apoptosis (ESCC), esophageal squamous cell carcinoma, mTOR/p70S6K pathway, PSMD7, tumorigenesis

## Abstract

PSMD7, a 19S proteasome subunit, is overexpressed in most carcinoma cells. It forms a dimer with PSMD14 that functions in the removal of attached ubiquitin chain. However, there is little knowledge about the cellular mechanism of PSMD7 and its exact biological function, especially in cancer cells. In this study, we explored the role of PSMD7 in proliferation, cell cycle, apoptosis, and proteasomal proteolysis in the esophageal squamous cell carcinoma (ESCC) cell line EC9706. Our results showed that PSMD7 was highly expressed in ESCC cells. Downregulation of PSMD7 by lentivirus‐mediated shRNA led to decreased proliferation, increased cell apoptosis, and reduced proteasomal function. Notably, lower expression level of mTOR and p70S6K and suppressed activity of mTOR/p70S6K pathway were detected after PSMD7 downregulation. By contrast, increased expression of p‐mTOR^Ser2448^ and p‐p70S6K^Thr421/Ser424^ was discovered upon PSMD7 overexpression in Het‐1A cells. Furthermore, PSMD7 downregulation contributed to decelerated tumor growth, inhibition of proteasomal function, induced cell apoptosis and attenuated activity of mTOR/p70S6K pathway *in vivo*. These findings suggest that PSMD7 and the mTOR/p70S6K pathway may be a promising candidate for developing therapies for ESCC.

**Abbreviations**
ESCC: esophageal squamous cell carcinoma
shRNA: short hairpin RNA
TGI: tumor growth inhibition rate
TV: tumor volume

Carcinoma of the esophagus is one of the most commonly diagnosed malignant cancers, ranking the eighth cancers worldwide with highly lethal rate and rapidly rising trend 1. In particular, the morbidity of esophageal squamous cell carcinoma (ESCC) remains high in northern China, which accounts for about 90% of the total esophageal cancer cases and possibly develops resistance to chemotherapeutic drugs 2. Chemical proteasomal inhibitors improve the survival rate of patients through strengthening the toxicity to cancer cells and increasing durable clinical response 3. It has been demonstrated that proteasome inhibitor bortezomib has anti‐proliferative effect not only to a large number of cancer cells, such as lung, breast, prostate, pancreas, and colon and melanoma cell lines, but also to xenograft models of solid tumors 4, 5, 6. However, disappointing results were yielded in clinical trials of solid tumors, no matter bortezomib used as single treatment or combined with other chemotherapy drugs, radiation therapy, immunotherapy, or other new targeting drugs in treatment 7, 8, 9. Thereafter, further investigation in the molecular mechanisms underlying the carcinogenesis and progression of ESCC and potential therapeutic targets becomes more and more urgent.

Accumulated studies in recent years have demonstrated that some deubiquitinases have abnormal expressions in tumors and participate in tumor development, which may be used as potential targets for drug therapy 10. The encoded deubiquitinases in human genome account for nearly 100 until now. It has been reported that four deubiquitinases (USP14, UCHL5/Uch37, PSMD7/RPN8, and PSMD14/RPN11) are related to proteasome function of removing or editing the ubiquitinated proteasome substrates 11. Among four deubiquitinases, PSMD7 and PSMD14 are non‐ATPase subunits of 19S lid subcomplex with Mpr1‐Pad1‐N‐terminal (MPN) domain, followed by long C‐terminal helices JAMM domain. PSMD14 has been reported to function in the removal of attached ubiquitin chain, which is a precondition for efficient substrate translocation through the narrow axial pore 12. Research on the proteasome holoenzyme revealed that PSMD14 and PSMD7 can form a dimer, which is locating directly above the central pore and leading the degraded substrate enter into the N‐terminal‐domain ring of the base ATPases 13.

In many types of tumor cells, in addition to the enhancement of the signaling pathways upstream to ubiquitin‐proteasome system, the activity of proteasome and expression of proteasomal subunits are abnormal 14, 15, 16. PSMD7 has been reported as a cancer‐associated signature in a variety of tumors, such as breast 17, 18, prostate 19, and bladder cancers 20. However, little knowledge about the cellular biological role of PSMD7 is known, and further research is needed to clarify its exact function in normal cells, particularly in cancer cells. Recent study has shown that inhibition of proteasomal activity induced cytotoxicity through the downregulation of ERK and Akt/mTOR signaling 21. Proteasome inhibitor MG132 caused the dephosphorylation of p70S6 kinase (p70S6K) and mTOR in a dose‐ and time‐dependent manner 22. The mTOR/p70S6K pathway is highly activated in ESCC, and its inhibition by rapamycin suppressed the phosphorylation of major downstream effectors, p70S6K and 4E‐BP1 23. Therefore, we supposed that PSMD7 was involved in the regulation of activity of mTOR/p70S6K pathway. Therefore, we hypothesized that PSMD7 maybe probably involved in the maintenance of proteasomal function in cancer cells.

In this study, the expression of PSMD7 in three ESCC cell lines was examined. In addition, its role on proliferation, cell cycle, cell apoptosis, and mTOR/p70S6K pathway was investigated using shRNA lentivirus‐mediated PSMD7 knockdown. Furthermore, we also found that PSMD7 knockdown *in vivo* suppressed the tumor growth using ESCC tumor models. Our results showed that PSMD7 is overexpressed in all three ESCC cells. Knockdown of PSMD7 showed inhibited proliferation and enhanced apoptosis, which is through mTOR/p70S6K signaling pathway. Taken together, PSMD7 exhibits pro‐cancer characteristics, which suggests PSMD7 could be a potential molecular target for ESCC therapy.

## Materials and methods

### Cell culture

Nonmalignant esophageal cell line Het‐1A was maintained in Dulbecco's modified Eagle's medium supplemented with 10% FBS (HyClone, USA). Human HeLa 229 cells and ESCC cell lines (EC9706, EC1 and Eca109 cells) were cultured in RPMI 1640 media (HyClone, USA) supplemented with 10% FBS and 1% L‐glutamine. All cells were grown at 37 °C humidified atmosphere with 5% CO_2_.

### Western blot

Total proteins of Het‐1A, HeLa 229, EC9706, and Eca109 were isolated by Total Protein Extraction Kit (Solarbio, China), respectively, followed by the detection of PSMD7 protein expression. Protein samples were subjected to SDS/PAGE gels and transferred to polyvinylidene fluoride membrane (Millipore, USA), followed by incubation with anti‐PSMD7 antibody (Santa Cruz Biotechnology, USA), anti‐GADPH antibody (Abgent, USA), anti‐cleaved caspase‐3 antibody (Abgent, USA), anti‐PARP antibody (Abgent, USA), anti‐ubiquitin antibody (Santa Cruz Biotechnology, USA), anti‐mTOR (Cell Signaling Technology, USA), p‐mTOR (Cell Signaling Technology, USA), p70S6K (Cell Signaling Technology, USA), and p‐p70S6K (Cell Signaling Technology, USA). Membranes were incubated with horseradish peroxidase‐labeled secondary antibodies (Boster, China) and detected by Enhanced Chemiluminescent Kit (Santa Cruz Biotechnology, USA).

### Immunofluorescence Staining

Cells were fixed by 4% paraformaldehyde and permeabilized by 0.1% saponin. Samples were blocked by 1% BSA and incubated with anti‐PSMD7 antibody (Santa Cruz Biotechnology, USA) and anti‐ubiquitin antibody (Santa Cruz Biotechnology, USA). Cell samples were visualized with the corresponding DyLight 649‐conjugated affinipure goat anti‐rabbit IgG (Earthox, USA). Cell nucleus was stained by incubation with 4′, 6‐diamidino‐2‐phenylindole (DAPI) for 10 min. Stained cells were imaged by IX71 inverted fluorescence microscope (Olympus Optical, Japan).

### Proteasomal activity assay

The cellular 26S proteasomal activity was evaluated by an *in vitro* peptide hydrolysis method using β5‐selective fluorogenic substrate succinyl‐Leu‐Leu‐Val‐Tyr‐7‐amido‐4‐methylcoumarin (Merck, Germany) as described previously 24. For some experiments, EC9706 cells were transfected with negative control shRNA lentivirus or PSMD7 shRNA lentivirus, and then, stable transfected cell populations were selected and maintained in puromycin culture medium.

### PSMD7 knockdown by lentivirus‐delivered RNA interference

Four short hairpin RNA (shRNA) sequences (Table 1) and one negative control scramble sequence (TTCTCCGAACGTGTCACGT) were designed and synthesized. The hairpin‐pGV112 vector was constructed containing a short hairpin RNA (shRNA) sequences. The lentivirus was packaged using the pHelper 1.0, pHelper 2.0, and pGV112 plasmids, as well as Lipofectamine 2000 (Invitrogen, USA), and then used to infect 293T cells. Lentivirus supernatant was collected 48 h later and used to infect EC9706 cells with polybrene (Sigma‐Aldrich, USA), followed by selection by puromycin (Thermo Scientific, USA).

**Table 1:** Targeting sequence of PSMD7 RNAi

NO.	Target Seq	CDS	GC%
LV‐PSMD7‐RNAi1	TGCACAACCTCATCAACAA	133…1107	42.11
LV‐PSMD7‐RNAi2	TGAGGAAGTTGGAGTTGAA	133…1107	42.11
LV‐PSMD7‐RNAi3	CATCAACGAACTCATGAAA	133…1107	36.84
LV‐PSMD7‐RNAi4	TGACAAAGACGATTCTGTA	133…1107	36.84

### Overexpression of PSMD7 in Het‐1A cells

The cDNA of PSMD7 was amplified by PCR and inserted into vector pEGFP‐N1 (Clontech, USA) to obtain a recombinated vector pEGFP‐N1‐PSMD7. EC9706 cells were seeded at a density of 2 × 10^6^ cells/mL followed by transfection with 2.5 μg of pEGFP‐N1‐PSMD7 and 5 μL of Lipofectamine 2000 (Invitrogen, USA) according to the instructions. Meanwhile, empty vector pEGFP‐N1 was transfected to EC9706 cells using as negative control.

### Cell proliferation and apoptosis analysis

Cells were seeded in 96‐well flat‐bottomed plate and cultured overnight, followed by detection of cell proliferation using CCK‐8 kit (Beyotime Inst Biotech, China). Cell apoptosis was detected using Annexin V‐APC/7‐AAD Apoptosis Detection Kit (KeyGEN Biotech, China) and analyzed by flow cytometry (Becton Dickson, USA). Annexin V^+^/7‐AAD^−^ and Annexin V^+^/7‐AAD^+^ cells were, respectively, considered as early and late apoptotic cells.

### Caspase activity assay

Caspase‐3 Fluorometric Assay Kit (BioVision, USA) was used to detect the caspase activity according to manufacturer's instructions. Cells were stained with Ac‐DEVD‐AFC substrate at 37 °C for 1 h. Tumor tissue sample was homogenized to generate tissue lysate and then followed the kit procedures. Fluorescence was generated from the cleavage of DEVD peptide and was measured by FACSCalibur flow cytometer (Becton Dickinson, USA).

### Animals and treatments

BALB/c nude mice (four‐ to six‐week‐old) were purchased from Hunan SJA Laboratory Animal Co., China, and raised under specific pathogen‐free (SPF) condition. All animal studies were carried out in compliance with the National Institutes of Health Guide for care and use of laboratory animals. Mice were housed four or five per cage in standard feeding condition.

EC9706 cells, negative shRNA control lentivirus, and PSMD7‐shRNA lentivirus‐transfected EC9706 cells were inoculated into the right flank of mice with 2 × 10^6^ cells each. All animals were inspected daily, and tumor volume (TV) was measured every 2 days. Tumor volume was calculated in accordance with the formula: V = 0.5AB^2^, in which A and B, respectively, represent the long and short diameter of tumor measured with vernier caliper. Mice were sacrificed 2 weeks later. Tumor inhibition is calculated as [(tumor volume of control group ‐ tumor volume of experimental group)/tumor volume of control group] ×100%.

Tumor tissues were separated into two parts: One part was rapidly put into liquid nitrogen and frozen for western blot and caspase activity analysis, and the other part was fixed with 10% formaldehyde and embedded by paraffin for immunohistochemical analysis.

### Immunohistochemistry

The paraffin‐embedded tumor tissue blocks were processed to obtain 5‐μm‐thick histologic sections. Samples were incubated with primary antibodies, anti‐cleaved caspase‐3 antibody (Abgent, USA), and anti‐ubiquitin antibody (Santa Cruz Biotechnology, USA) overnight at 4 °C, followed by staining with biotinylated antibody coupled to HRP‐linked streptavidin/biotin complex (Santa Cruz Biotechnology, USA). Then, the samples were visualized by DAB staining.

### Statistical analysis

Data were shown as mean ± SD from three independent experiments with significance calculated by Students’ *t*‐test. The *P* value < 0.05 was considered as statistically significant.

## Results

### PSMD7 expression in ESCC cell lines

**Figure 1:**
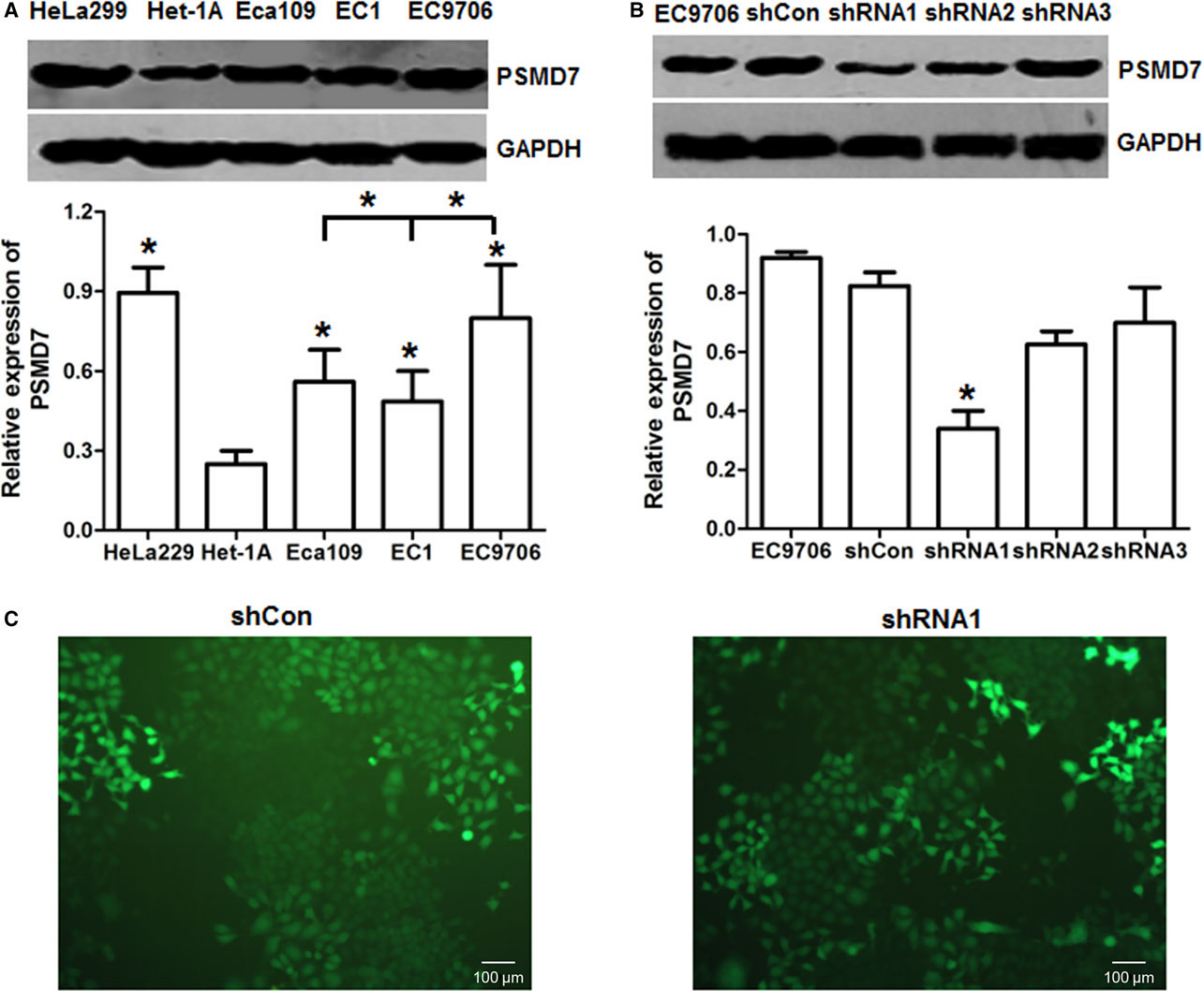
PSMD7 was overexpressed in esophageal squamous cell carcinoma (ESCC) cell lines. (A) Western blot results of PSMD7 in ESCC cell lines with GAPDH as internal control. (B) Lentivirus shRNA vectors targeting PSMD7 were constructed and used to infect EC9706 cells. The protein expression level of PSMD7 was examined by western blot 72 h after lentivirus transfection. *P <* *0.05 by Student’ s *t*‐test. Error bars represent mean ± SD derived from three independent experiments. (C) The lentivirus‐transfected cells were selected by puromycin to obtain stable cell populations. The transfection efficiency was indicated by GFP fluorescent signal (×100).

To explore PSMD7 expression level in cancer cells, the relative protein expression of PSMD7 was examined in nonmalignant esophageal cell line Het‐1A and ESCC cell lines. Results showed that PSMD7 was highly expressed in three ESCC cell lines and HeLa 299 cell line (Fig. 1A). The expression of PSMD7 showed statistical difference between the three ESCC cell lines and Het‐1A cells. The protein level of PSMD7 was higher changed by ~ 3.2 times in EC9706 cells (*P *<* *0.05), ~ 1.96 times in EC1 cells (*P *<* *0.05), and ~ 2.24 times in Eca109 cells (*P *<* *0.05) than Het‐1A cells (Fig. 1A). The protein expression level of PSMD7 in EC9706 cells was high and was employed in our subsequent studies. Therefore, we found PSMD7 is differently expressed in cancer cell lines.

### PSMD7 knockdown inhibits proliferation and induces apoptosis in ESCC cell line

We have found that PSMD7 was highly expressed in EC9706 cells. To investigate affection by PSMD7 knockdown on malignant cell proliferation, lentivirus shRNA vectors targeting PSMD7 were constructed and used to infect EC9706 cells. The protein expression level of PSMD7 was decreased by 63% in shRNA1 lentivirus‐transfected EC9706 cells compared with the control (Fig. 1B), so PSMD7 shRNA1 lentivirus can inhibit the expression of PSMD7 in EC9706 cells. In the meantime, the lentivirus‐transfected cells were selected by puromycin to obtain stable knockdown cell populations (Fig. 1C). Together, we obtained transient and stable PSMD7 knockdown EC9706 cells.

**Figure 2:**
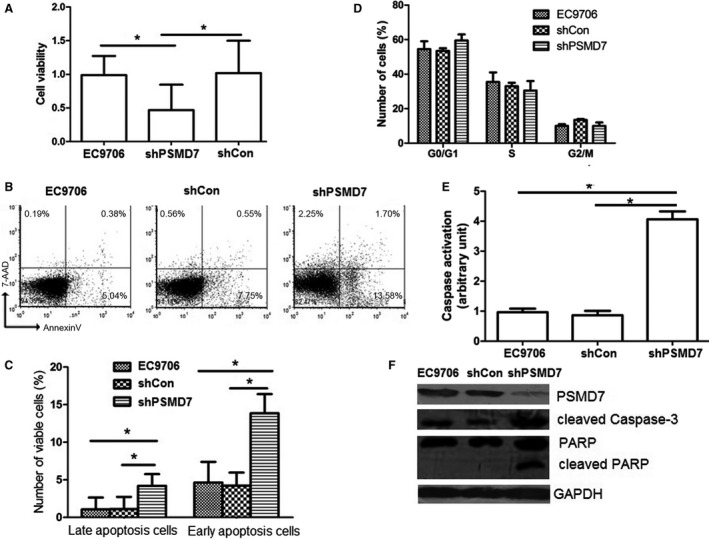
Effect of PSMD7 knockdown on proliferation, cell cycle, and apoptosis in EC9706 cells. (A) Cells were subjected to CCK‐8 assay for the determination of cell proliferation. shCon: EC9706 cells transfected with control lentivirus; shPSMD7: EC9706 cells transfected with shRNA1 lentivirus targeting PSMD7. *P <* *0.05 by Student’ s *t‐*test. Error bars represent mean ± SD derived from three independent experiments. (B) One representative of flow cytometry scatter plots showed Annexin V‐FITC staining and 7‐AAD from three independent experiments. (C) The numbers represent the percentage of early apoptotic cells (lower right quadrant) and late apoptotic cells (upper right quadrant) are shown. (D) Cell cycle distribution data were shown in PSMD7 knockdown in C9706 cells. (E–F) Caspase activation assay (E) and immunoblot assay of cleaved by caspase‐3 and PARP (F) in EC9706 cells were performed. Results are presented as the mean ± SD based on three independent experiments. *P <* *0.05 by Student’ s *t‐*test.

Next, cell proliferation capability of parental EC9706, negative control shRNA‐transfected (shCon), and PSMD7‐targeted shRNA‐transfected (shPSMD7) EC9706 was explored by CCK‐8 assay. Knockdown of PSMD7 considerably decreased the cell viability with ~ 47% of shPSMD7 group compared to shCon group (*P *< 0.05) (Fig. 2A), suggesting that inhibition of PSMD7 decreased the survival of EC9706 cells. Whether PSMD7 knockdown in EC9706 cells had impacts on apoptosis and cell cycle distribution was investigated by flow cytometry. Results showed that inhibition of PSMD7 in EC9706 cells caused cell apoptosis with significantly increased numbers of early apoptosis cells and late apoptosis cells (*P *< 0.05) (Fig. 2B,C), but made no difference on cell cycle distribution (*P *>* *0.05) (Fig. 2D). The caspase‐3 activity was quantified through detecting the fluorogenic response as a result of DEVD peptide cleavage (Fig. 2E). PSMD7 knockdown raised caspase‐3 activity compared with the control (*P < *0.05), which was further confirmed by the increased cleavage of caspase‐3 in shPSMD7 group (Fig. 2F). As a marker for cell apoptosis, PARP is cleaved by caspase‐3 and facilitates disassembling of the cellular components. A prominent cleaved PARP band was detected in PSMD7 knockdown tumor cells, whereas no PARP cleavage band was observed in control groups (Fig. 2F). In conclusion, PSMD7 knockdown inhibits proliferation and induces apoptosis in ESCC cells.

### Decreased proteasomal activity after PSMD7 knockdown

**Figure 3:**
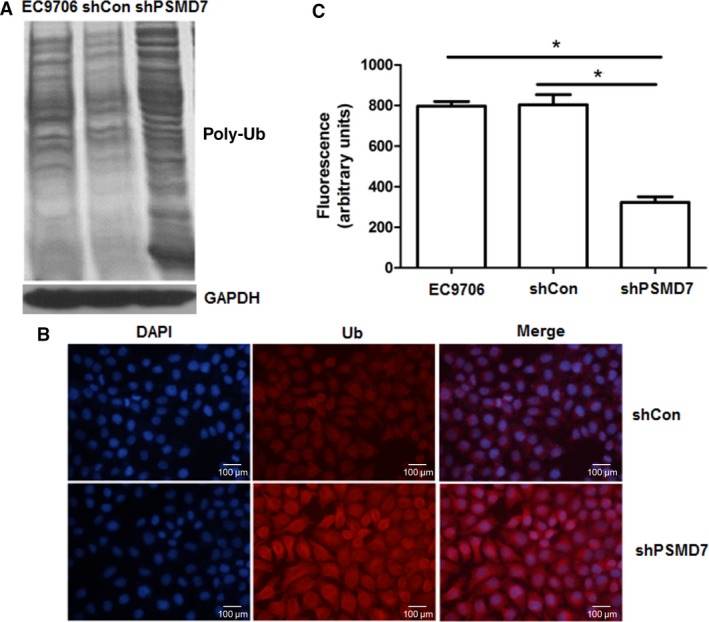
Knockdown of PSMD7 affected the ubiquitin‐proteasome pathway. (A) Western blots of ubiquitin from cell lysate showed the level of ubiquitinated protein in PSMD7 normal and knockdown condition. (B) Immunofluorescence of ubiquitin conjugates after lentivirus transfection (×200). (C) The proteolytic capability of 26S proteasome was measured by an *in vitro* peptide hydrolysis method using the β5‐selective fluorogenic substrate succinyl‐Leu‐Leu‐Val‐Tyr‐7‐amido‐4‐methylcoumarin. *P <* *0.05 by Student’ s *t‐*test. Error bars represent mean ± SD derived from three independent experiments.

As ubiquitin‐proteasome system plays an important role in apoptosis, western blot of ubiquitin from cell lysate showed that PSMD7 knockdown resulted in a remarkable increased level of ubiquitinated protein (Fig. 3A). Also, stronger fluorescence of ubiquitin and reduced proteasomal activity (*P < *0.05) were observed in PSMD7 knockdown cells in comparison with control (Fig. 3B,C). So, the increased total level of ubiquitinated protein and decreased proteasomal activity supported that knockdown of PSMD7 was involved in the disruption of the ubiquitin‐proteasome pathway.

### Effect of PSMD7 downregulation on the mTOR/p70S6K pathway

**Figure 4:**
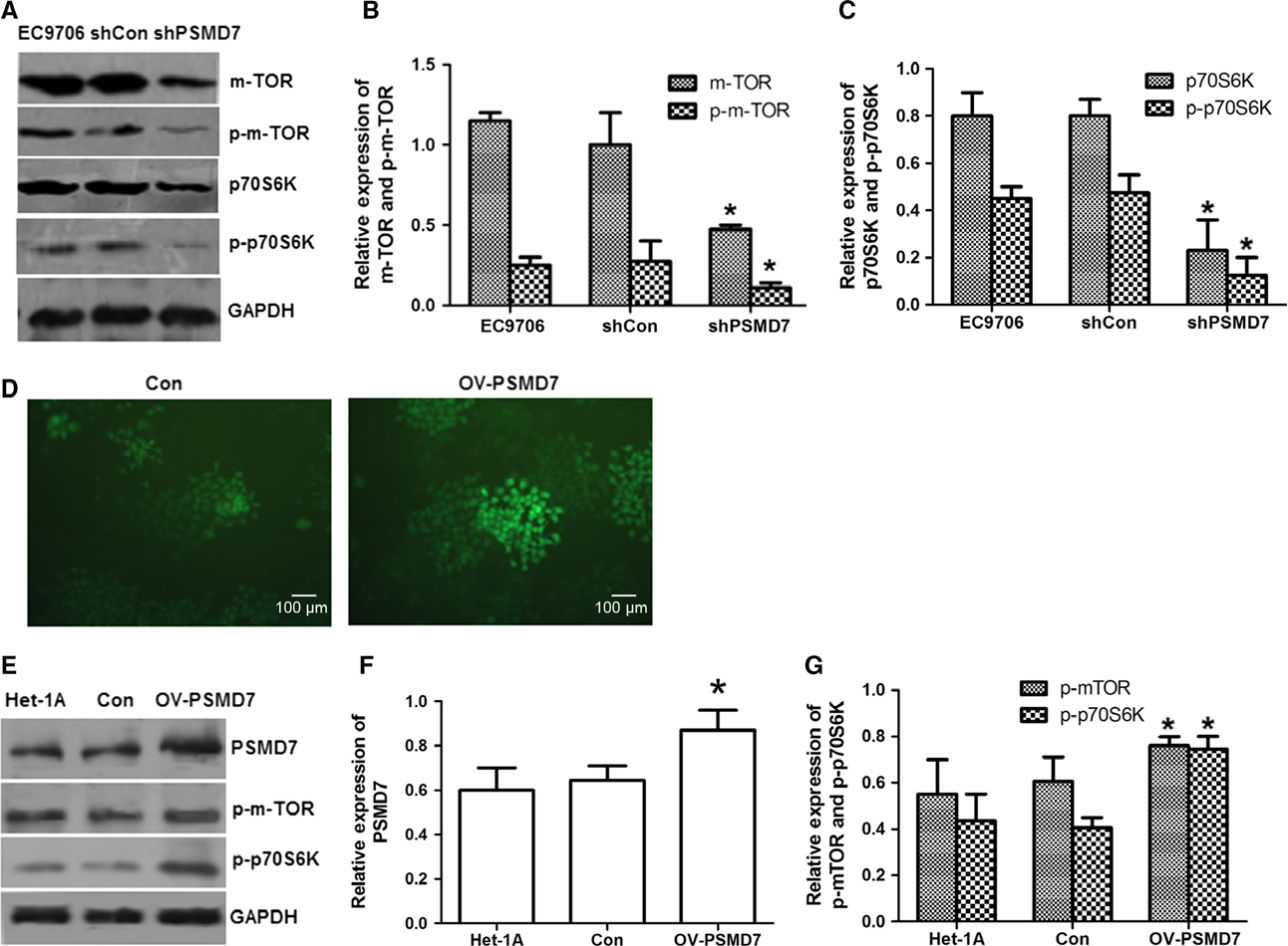
Effects of PSMD7 on the activity of mTOR/p70S6K pathway in EC9706 cells and Het‐1A cells. (A) Western blot results of mTOR, p‐mTOR, p70S6K, and p‐p70S6K with GAPDH as internal control. (B and C) The expression of mTOR, p‐mTOR, p70S6K, and p‐p70S6K was analyzed by densitometry using ImageJ software and plotted from three independent experiments. (D) PSMD7 was overexpressed in Het‐1A cells by transfection with the constructed vector pEGFP‐N1‐PSMD7 (OV‐PSMD7) using empty vector as control (Con). The green fluorescence indicated the transfected cells (×100). (E) Western blot results of PSMD7, p‐mTOR, and p‐p70S6K with GAPDH as internal control. (F and G) The expression of PSMD7, p‐mTOR, and p‐p70S6K was analyzed by densitometry using ImageJ software and plotted from three independent experiments. *P <* *0.05 by Student’ s *t‐*test.

To explore the potential mechanisms underlying the inhibited proliferation and increased cell apoptosis due to PSMD7 inhibition, the protein expression levels of mTOR and p70S6K were investigated by western blot (Fig. 4A). Compared with the control groups, the expression levels of mTOR and p70S6K were reduced in shPSMD7 group, ~ 52.5% (*P *<* *0.05) and ~ 71.3% (*P *<* *0.05), respectively (Fig. 4B,C). Moreover, the protein level of p‐mTOR^Ser2448^ and p‐p70S6K^Thr421/Ser424^ was reduced by ~ 60.0% (*P *<* *0.05) and ~ 73.7% (*P *<* *0.05), respectively (Fig. 4B,C), suggesting the suppressed activity of mTOR/p70S6K pathway in PSMD7 knockdown cells. Moreover, PSMD7 was overexpressed in cells of Het‐1A by transfection with the constructed vector pEGFP‐N1‐PSMD7 (Fig. 4D). Results showed that the protein level of PSMD7 was increased to ~ 1.45 times compared with control (*P *<* *0.05) (Fig. 4E,F). The protein level of p‐mTOR^Ser2448^ and p‐p70S6K^Thr421/Ser424^ in PSMD7 overexpressing Het‐1A cells was raised to ~ 1.26 times (*P *<* *0.05) and ~ 1.84 times (*P *<* *0.05), respectively, compared with controls (Fig. 4G).

### Inhibited tumor growth *in vivo* by PSMD7 knockdown

**Figure 5:**
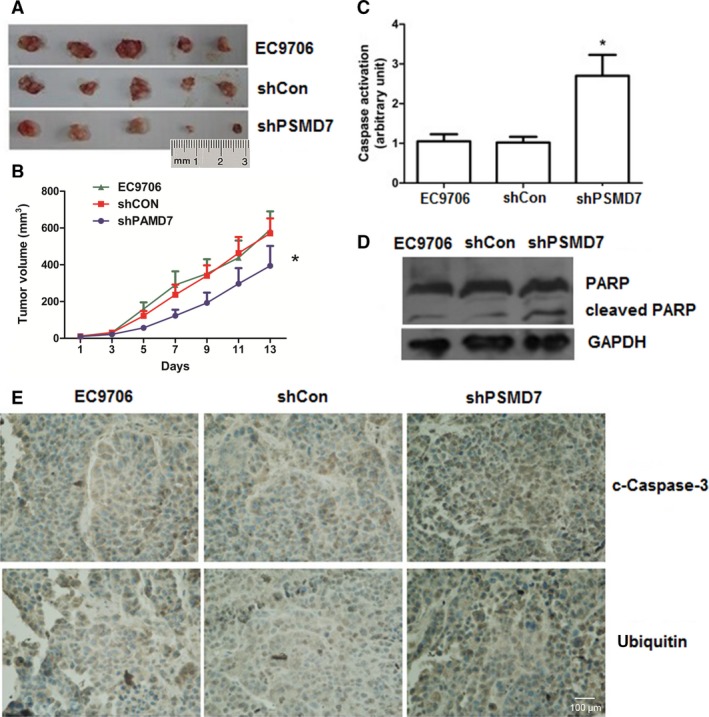
PSMD7 knockdown regulates tumor growth in an ESCC mouse model. (A) Tumor tissue retrieved from ESCC xenografts (*n* = 5). (B) The mean tumor volume of ESCC xenografts was calculated and plotted. (C–E) Caspase activation (C), western blot for PARP cleavage (D), and immunohistochemical staining (E) of tumor section obtained from mice are shown. Error bars represent mean ± SD derived from three independent experiments. **P <* *0.05.

As the apoptosis was enhanced and the proteasomal activity was inhibited in the *in vitro* experiments, the potential effect of PSMD7 knockdown on the inhibition of ESCC tumorigenesis *in vivo* was subsequently tested. The tumor‐bearing mice models were established using EC9706 cells and PSMD7 shRNA lentivirus infected EC9706 cells, and tumor volumes were monitored every 2 days. Tumors were excised at the end of the experiment (Fig. 5A). Tumor volumes were calculated and plotted (Fig. 5B). Our results showed that the growth rate of xenograft in shPSMD7 group was decreased by comparison with control groups (*P *<* *0.05), with tumor growth inhibition rate (TGI) of shPSMD7/shCon as 58.3% at day 14 after inoculation (Fig. 5B). Summary, PSMD7 knockdown induces impaired tumor growth *in vivo*.

As shown in Fig. 5C, the activity of caspase‐3 in shPSMD7 group was higher than that in control groups (*P *<* *0.05). Meanwhile, it was associated with increased expression of cleaved PARP and stronger staining of cleaved caspase‐3 after PSMD7 inhibition (Fig. 5D), suggesting that cell apoptosis was induced by PSMD7 knockdown *in vivo*. The expression intensity of ubiquitin in xenograft of nude mice was analyzed by immunohistochemical assay. High density of ubiquitinated protein was found in shPSMD7 group (Fig. 5E), which confirmed the inhibitory role of PSMD7 in ubiquitin‐proteasome system *in vivo*.

**Figure 6:**
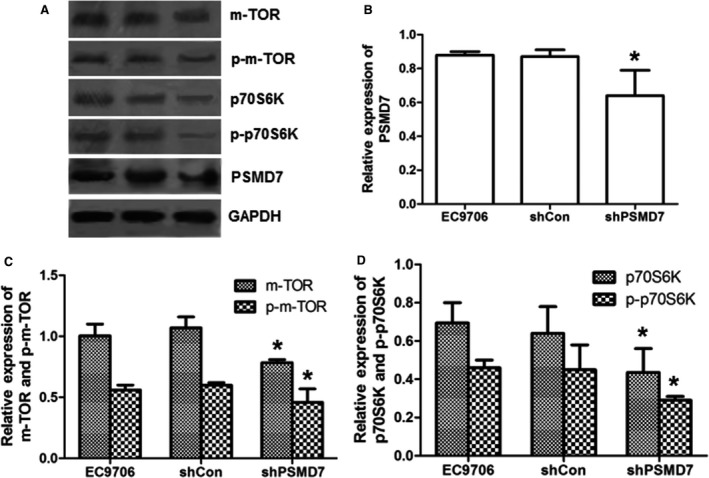
Effect of PSMD7 knockdown on the activity of mTOR/p70S6K pathway *in vivo*. (A) Western blot results of PSMD7, mTOR, p‐mTOR, p70S6K, and p‐p70S6K normalized with GAPDH expression. (B–D) The expression of PSMD7, mTOR, p‐mTOR, p70S6K, and p‐p70S6K was analyzed by densitometry using ImageJ software and plotted from three independent experiments. *P <* *0.05 by Student’ s *t‐*test.

Based on the inhibited activity of the mTOR pathway in PSMD7 knockdown ESCC cell line, whether the anti‐cancer effect of PSMD7 knockdown *in vivo* through mTOR/p70S6K inhibition was evaluated. The expression of mTOR and p70S6K, and the phosphorylation status of them in xenograft were examined (Fig. 6A,B). The expression of PSMD7 is significantly reduced in shPSMD7 group (*P *<* *0.05). PSMD7 knockdown blocked the expression levels of mTOR and p70S6K *in vivo*, reduced by 33% and 37%, respectively (*P *<* *0.05) (Fig. 6C,D). The protein levels of p‐mTOR^Ser2448^ and p‐p70S6K^Thr421/Ser424^ were also decreased by 18% (*P *<* *0.05) and 36% (*P *<* *0.05), respectively (Fig. 6C,D). These data suggest that inhibition of PSMD7 suppressed the activity of mTOR/p70S6K *in vivo*.

## Discussion

In this study, we found the high expression of PSMD7 and elevated proteasomal activity in ESCC cells. Recent studies have shown that siRNA targeting PSMD7 increased fluorescence level of an ubiquitin‐dependent cytoplasmic GFP reporter similar to proteasome inhibitor MG132 treatment leading to cell death 25. In our study, we found that PSMD7 have the similar cellular effect similar with proteasome inhibitor. It is interesting that we, at the first time, reported that PSMD7 knockdown resulted in decreased proliferation and enhanced apoptosis in ESCC cell lines. Moreover, PSMD7 knockdown cells had higher caspase‐3 activity and strengthened the cleavage of caspase‐3 compared with control cells (ineffective shRNA). Also, the cleavage of PARP band was detected in PSMD7 knockdown tumor cells, but not in control groups. These data provided strong evidences that PSMD7 impairment induced apoptosis of ESCC cell lines.

Structural research on PSMD7 revealed that the JAMM domain is a metal‐free structure without isopeptidase activity 26, suggesting that it may not incise the poly‐ubiquitinated protein chain directly. However, PSMD7 was not detected as an individual subunit but presented in heteropolymer, in which it occupied a central position in an independent subcomplex isolated from 19S proteasome 27. The depletion of PSMD7 may affect the assembly of 19S lid subcomplex and eventually decrease the ability of recognition to poly‐ubiquitinated chain and cleavage of the degraded protein from ubiquitinated chain. Here, we showed that a remarkable increase in ubiquitinated protein levels, elevated fluorescence for ubiquitin, and reduced proteasomal activity were observed in PSMD7 knockdown ESCC cells, suggesting that PSMD7 participated in the maintenance of normal proteasome function. It may also suggest that the decreased proteasome activity was due to the abnormal function of 19S proteasome caused by PSMD7 knockdown.

The mTOR/p70S6K pathway exerts critical function in cell proliferation and is regulated by other protein 28, 29. In ESCC cell lines, mTOR/p70S6K pathway is activated *via* phosphorylation at multiple serine and threonine residues 30. MG132 has been shown to cause the dephosphorylation of p70S6K/mTOR and induce cell autophagy 22. Inhibition of PSMD7 not only reduced the expression of mTOR and p70S6K, but also decreased the expression of p‐mTOR^Ser2448^ and p‐p70S6K^Thr421/Ser424^, whereas the augmented expression of phosphorylation of mTOR and p70S6K was detected after PSMD7 overexpression in Het‐1A cells. Our results showed that the effect of PSMD7 depletion in ESCC cell lines was similar to MG132 treatment, but the decreased phosphorylation residue of p70S6K was different, which are Thr421/Ser424 in PSMD7 knockdown and Thr389 in MG132 treatment. Our results indicated that the cellular function exhibited by PSMD7 inhibition in ESCC cell lines possibly had close relationship to the mTOR/p70S6K pathway.

In ESCC xenografts bearing mice with repression of PSMD7, we also proved that *in vivo* ability of PSMD7 inhibition suppresses tumor growth and induces apoptosis. Knockdown of PSMD7 deferred tumor growth in comparison with controls. The *in vivo* experimental results were consistent with those of the *in vitro* experiments, as the caspase activity and the level of cleaved PARP and ubiquitinated protein were significantly elevated after PSMD7 inhibition. Furthermore, PSMD7 inhibition restrained the mTOR pathway by reducing the phosphorylation of mTOR at Ser2448 and p70S6K at Thr421/Ser424 *in vivo*. Thus, we concluded that the suppression impact of PSMD7 on ESCC xenograft model may be *via* impeding the mTOR/p70S6K pathway.

From the above discussion, conclusion can be reached that PSMD7 downregulation contributes to inhibited proteasomal function and induced apoptosis in ESCC cell lines, which is likely to tightly associate with the mTOR/p70S6K pathway. Hence, operation of PSMD7 and mTOR/p70S6K pathway is a promising candidate target for therapy of ESCC.

## Author contributions

KS and DG conceived and designed the project, R‐lZ, KS, and LY acquired the data, KS and J‐zZ analyzed and interpreted the data, as well as wrote the article.
